# Navigating Diagnostic Challenges in Acute Coronary Syndrome: A Case of Bezold-Jarisch Reflex and Wellens Pattern

**DOI:** 10.7759/cureus.60323

**Published:** 2024-05-15

**Authors:** Nikita Singh, Pawel Borkowski, Shaunak Mangeshkar, Vibhor Garg, Bisrat H Adal

**Affiliations:** 1 Internal Medicine, Albert Einstein College of Medicine, Jacobi Medical Center, New York, USA

**Keywords:** wellens pattern, acute coronary syndrome, diagnostic challenges, cardiovascular disease, percutaneous coronary intervention, coronary angiography, electrocardiogram, bezold-jarisch reflex

## Abstract

Acute coronary syndrome (ACS) presents significant diagnostic challenges, particularly in cases with atypical presentations and complex clinical scenarios. Here, we describe the case of a 59-year-old man who presented with presyncope, bradycardia, hypotension, and later syncope, attributed to the Bezold-Jarisch reflex. Electrocardiographic findings suggested both inferior and anterior wall infarction, with dynamic changes in T-wave morphology further complicating the diagnostic process. Despite a type A Wellens' pattern indicating critical stenosis in the proximal left anterior descending (LAD) artery, coronary angiography revealed a complete thrombotic lesion in the proximal right coronary artery (RCA), necessitating urgent intervention. Despite the Wellens pattern indicating LAD involvement, RCA revascularization took precedence due to immediate thrombotic risk. This case underscores the diagnostic challenges associated with conflicting clinical manifestations in ACS and highlights the importance of individualized management strategies integrating advanced diagnostic modalities to optimize outcomes. Understanding the interplay of complex clinical presentations and employing a nuanced approach to management are crucial in effectively navigating ACS scenarios.

## Introduction

Acute coronary syndrome (ACS) is a leading cause of morbidity and mortality worldwide. According to data from the American Heart Association, an estimated 805,000 Americans annually experience an ACS event. Among these cases, 605,000 represent new attacks, while 200,000 are recurrent attacks [1]. Despite advances in management strategies, the diagnosis of ACS continues to pose significant challenges, particularly in patients presenting with atypical symptoms or complex clinical scenarios. While chest pain is the hallmark symptom of ACS, up to one-third of cases may present with atypical symptoms, such as dyspnea, nausea/vomiting, or syncope, making timely diagnosis and intervention crucial for improving patient outcomes [2].

The Bezold-Jarisch reflex is characterized by hypotension, bradycardia, and peripheral vasodilation and represents a complex interplay between cardiac physiology and autonomic regulation. Although possible physiologic roles of the Bezold-Jarisch reflex are thought to be in blood pressure regulation and homeostatic response to hypovolemia, it may also provoke notable hemodynamic shifts in the presence of myocardial ischemia [3]. Inferior wall myocardial ischemia is a known trigger of this reflex due to ventricular mechanoreceptor stimulation [4].

Concurrently, the recognition of the Wellens pattern on electrocardiogram (ECG) adds another layer of complexity. Wellens pattern indicates critical stenosis in the proximal left anterior descending (LAD) artery and heralding the risk of anterior wall myocardial infarction (MI) [5]. In this context, we present a compelling case of a 59-year-old man, offering a view into the intricate interplay of presentation attributed to the Bezold-Jarisch reflex within the context of inferior wall MI, and the diagnostic challenges posed by electrocardiographic findings. Through a comprehensive analysis of a unique case, this report aims to dissect the diagnostic challenges posed by contradictory findings in the context of ACS and tailoring individualized management.

## Case presentation

A 59-year-old man presented with a sudden onset history of presyncope. He described the development of “sweatiness” after his usual morning walk associated with light-headedness and loss of stool continence without loss of consciousness. The patient immediately reported stool incontinence to his son, who called the emergency medical services (EMS), who arrived approximately in 15 minutes. He reported similar episodes several years ago, especially during straining or after having a bowel movement. In the field, the EMS found the patient's blood pressure to be 72/44 mmHg with a heart rate of 52 beats per, which responded to 1 mg atropine and a fluid bolus of one liter of normal saline. Following the resuscitation efforts by EMS, on arrival to the emergency department, there was a notable improvement recorded in the vitals signs with a blood pressure increase to 126/76 mmHg and a heart rate normalization to 74 beats per minute, respiratory rate of 14 breaths per minute, and temperature of 98.2°F. Physical examination revealed an individual appearing his stated age, with a body mass index of 31.4 kg/m^2^ with no jugular venous distention, clear lung sounds, soft abdomen, regular heart sounds (with no murmurs, rubs, or gallops), and warm extremities. The patient had a medical history of hypertension and type 2 diabetes mellitus. However, he had stopped his diabetes and hypertension medications over the past several years. He reported consumption of alcoholic drinks once or twice a year and no history of smoking or recreational drug use. The patient's vital signs were stable at presentation to the emergency department, with a blood pressure of 126/76 mmHg and a heart rate of 74 beats per minute following resuscitation with 1 mg of atropine and one liter of fluid bolus by EMS. Although the patient had experienced an episode of witnessed syncope in the emergency department, there were no signs suggestive of shock or poor perfusion noted at that time.

In the emergency department, blood tests showed initial troponin T levels of 0.212 ng/mL (reference range: 0.000-0.090 ng/mL), creatine phosphokinase of 477 mcg/L, glycated hemoglobin of 9.8%, and thyroid-stimulating hormone of 0.827 uIU/mL.

**Figure 1:**
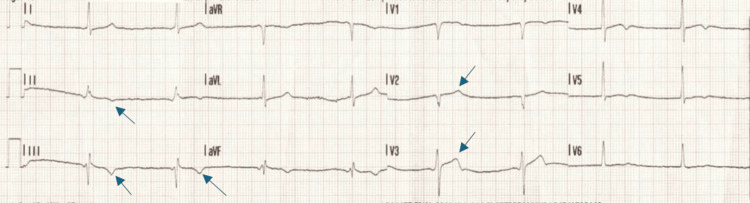
A standard 12-lead electrocardiogram recorded at a paper speed of 25 mm/s by emergency medical services shows sinus bradycardia with a heart rate of 51 beats per minute, T-wave inversions in inferior leads III and aVF and ST elevations in leads V2 and V3

**Figure 2:**
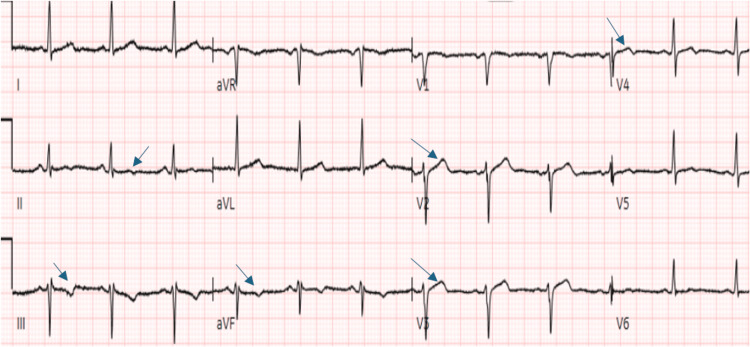
Electrocardiogram at presentation to the emergency department shows normal sinus rhythm with T-wave inversions in leads II, III, and aVF, as seen in the ECG recorded by the emergency medical services (Figure 1). ST elevation can now be seen in leads V2, V3, and V4

**Figure 3:**
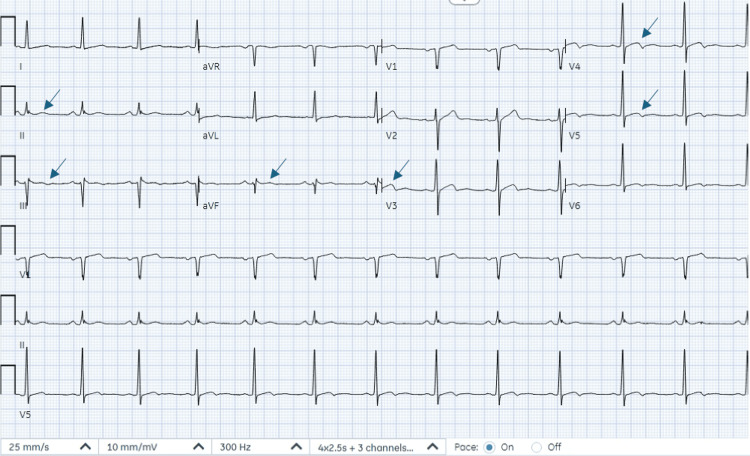
Electrocardiogram recorded 12 hours after presentation shows new subtle biphasic T waves in V3-V5, which may simulate the Wellens pattern. Improvement of T-wave inversions in lead III and resolution of T-wave inversions in leads II and aVF are also seen

The initial standard 12-lead electrocardiogram (ECG), recorded at a paper speed of 25 mm/s by emergency medical services, revealed sinus bradycardia, with a heart rate of 51 beats per minute, along with T-wave inversions in the inferior leads (II, III, aVF), suggestive of inferior wall ischemia and ST elevation in leads V2 and V3 (Figure 1). On arrival at the emergency department, another standard 12-lead ECG, recorded at a paper speed of 25 mm/s with an amplification of 10 mm/mV, showed normal sinus rhythm with T-wave inversions in leads II, III, and aVF, consistent with the ECG recorded by emergency medical services (Figure 1) and ST elevation in leads V2, V3, and V4, suggestive of anterior wall myocardial infarction (Figure 2). During the hospital course, a subsequent ECG recorded 12 hours after presentation showed new subtle biphasic T waves in V3-V5, along with improvement of T-wave inversions in lead III and resolution of T-wave inversions in leads II and aVF (Figure 3). Transthoracic echocardiography revealed mild hypokinesis of the basal to mid-anterior and anteroseptal wall.

The patient's clinical presentation, along with laboratory and electrocardiogram findings, prompted consideration of several differential diagnoses. These included acute coronary syndrome, vasovagal syncope (due to the witnessed syncope in the emergency department, supported by the patient's history of similar episodes during straining or bowel movements), and arrhythmia (as suggested by the bradycardia noted in the field along with syncopal episode). Notably, the T-wave inversions in precordial leads present in all serial ECGs may simulate Wellens pattern. The T-wave changes in the ECG and elevated cardiac biomarkers steered the diagnosis toward an ACS event.

The patient received an aspirin load of 162 mg from the EMS. Following initial evaluation in the ED, the acute coronary syndrome (ACS) protocol was initiated. The patient was given a clopidogrel load of 300 mg and started on an unfractionated heparin (UFH) infusion. The dosage and rate of the UFH infusion were carefully adjusted based on the patient's weight and coagulation parameter, activated partial thromboplastin time (aPTT), to achieve a goal aPTT of 50-70 seconds (corresponding to approximately 1.5-2.5 times the control value of aPTT) while minimizing the risk of bleeding complications. The patient was initiated on high-intensity statin therapy, specifically atorvastatin at a dose of 80 mg daily, and a beta-blocker, metoprolol tartrate, was initiated at a dose of 12.5 mg twice daily within 24 hours of presentation.

**Figure 4:**
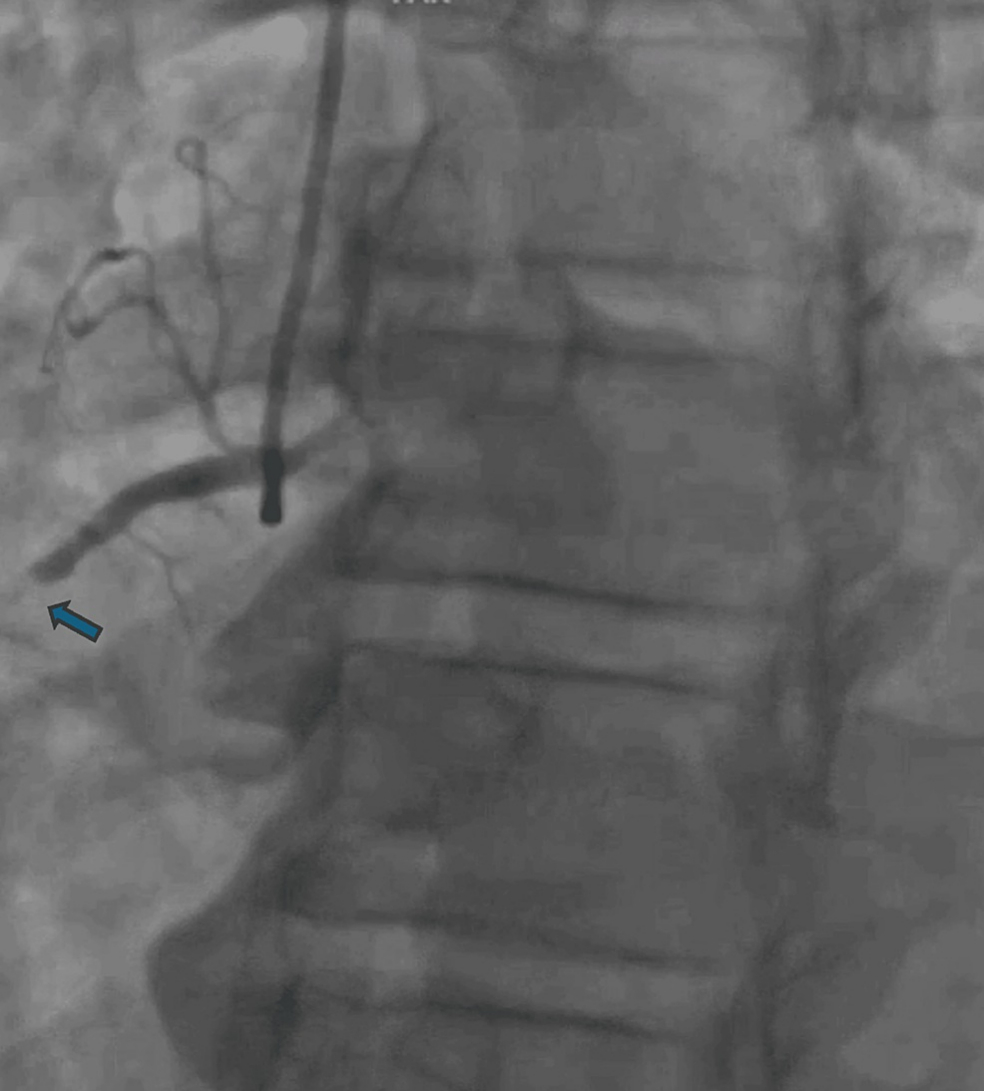
Complete thrombotic occlusive lesion in the proximal right coronary artery (RCA) with 100% stenosis

**Figure 5:**
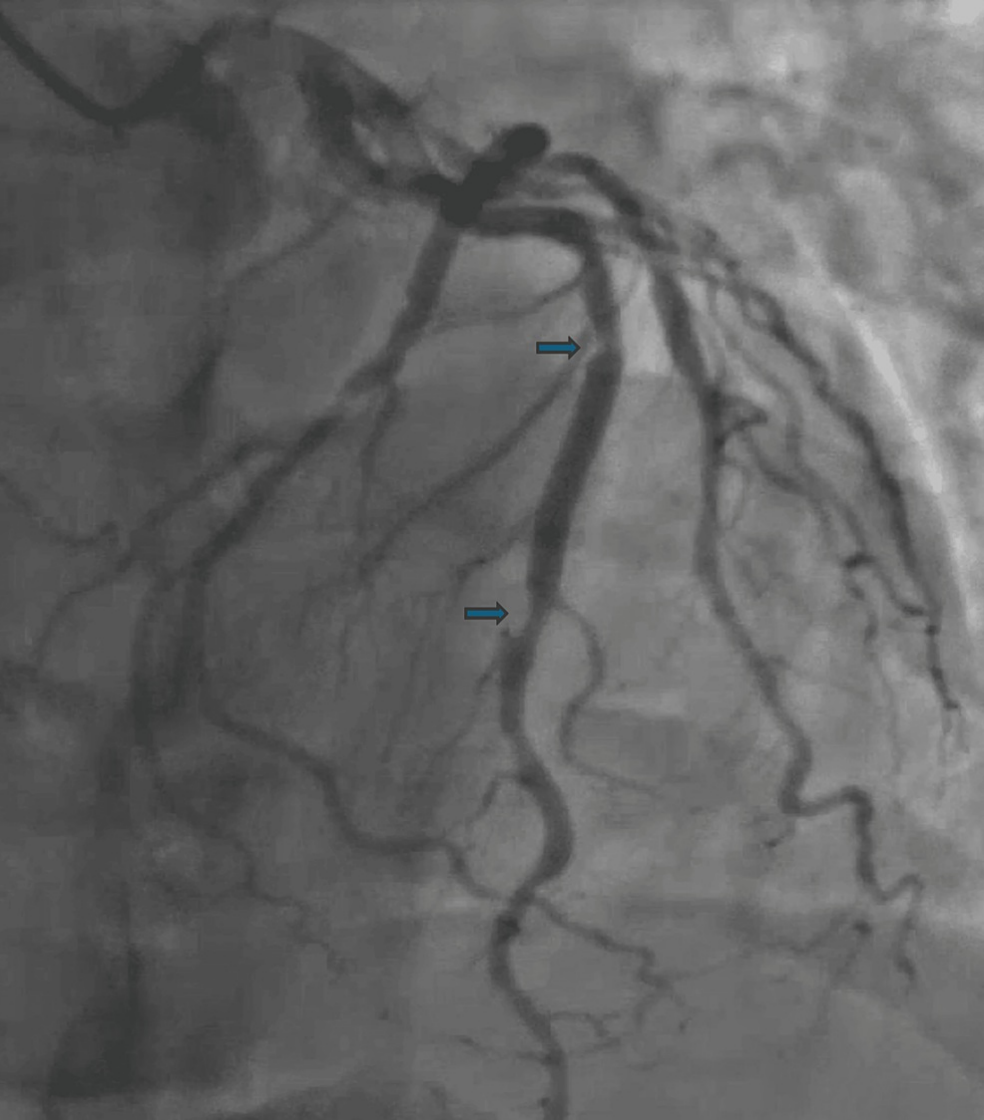
Severe diffuse disease in the mid- and distal left anterior descending (LAD) artery, with visually estimated 75% stenosis and TIMI-3 flow TIMI: Thrombolysis in Myocardial Infarction

**Figure 6:**
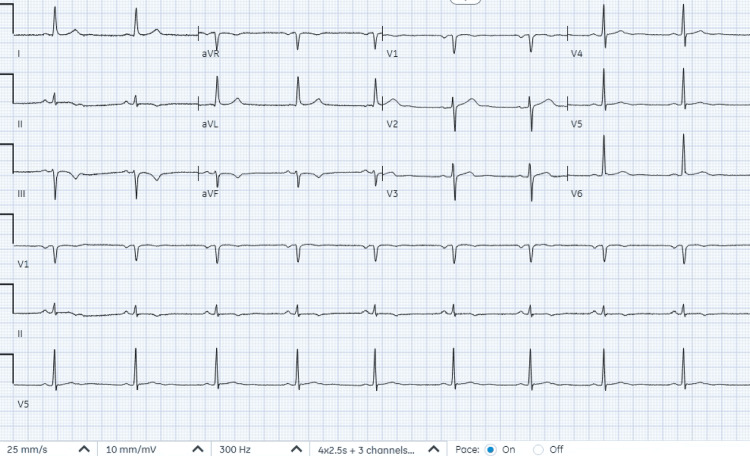
An electrocardiogram recorded post-revascularization shows sinus bradycardia, now with upright T waves in leads V2-V5

He underwent left heart catheterization (LHC) for both diagnostic and therapeutic purposes. LHC revealed a complete thrombotic lesion in the proximal right coronary artery (RCA), visually estimated as complete occlusion (100% stenosis) (Figure 4) with a pre-intervention thrombolysis in MI (TIMI) of 0. Intravascular ultrasound (IVUS) examination confirmed severe plaque burden and a concentric nature of the lesion, managed with IVUS-guided percutaneous coronary intervention (PCI) using one drug-eluting stent (DES). Subsequently, severe diffuse disease was observed in the mid-RCA after recanalization of the occluded proximal segment, also treated with IVUS-guided PCI using one DES. Additionally, severe residual disease was found in the distal LAD artery (Figure 5). The distal LAD artery exhibited a visually estimated 75% stenosis, with a pre-intervention TIMI of 3. IVUS assessment revealed a minimum luminal area (MLA) of 3.5 mm², indicative of significant stenosis and a concentric nature of the lesion. These findings suggest a critical condition in both arteries, with the proximal RCA lesion posing an immediate threat due to its thrombotic nature and complete occlusion. Meanwhile, the distal LAD lesion, although less severe in terms of visual stenosis, presents a substantial narrowing of the vessel lumen, as indicated by the MLA measurement. The decision to prioritize intervention on the RCA was based on the angiographic evidence of a complete thrombotic lesion in the proximal RCA, which posed an immediate threat and warranted urgent attention, despite the ECG findings suggesting LAD territory ischemia and the echocardiographic evidence of hypokinesis in the anterior wall. Post-revascularization ECG showed sinus bradycardia with non-specific T-wave abnormalities (Figure 6).

Post-procedure, the patient underwent gradual ambulation as part of the recovery process. A physical therapy evaluation confirmed the patient's functional independence, indicating that additional physical therapy services were not required at that time. The patient was advised to continue dual antiplatelet therapy with aspirin and clopidogrel for at least one year, in addition to maintaining strict control over blood pressure and glycemic levels. Upon discharge, the patient's medication regimen included high-intensity statin therapy with atorvastatin and lisinopril for blood pressure management. A beta-blocker was not prescribed on discharge as the patient had sinus bradycardia on post-revascularization ECG (Figure 6). Lifestyle modifications and a gradual return to normal activities were strongly encouraged to optimize cardiovascular health and promote overall health. Regular follow-up appointments were scheduled to monitor the patient’s progress and adherence to the therapeutic regimen.

## Discussion

The absence of typical chest pain and the presence of bradycardia, hypotension, and syncope due to the Bezold-Jarisch reflex in the setting of inferior wall ST-elevation MI, as evidenced by the initial clinical presentation of cardiogenic shock and the angiographic findings of a totally occluded right coronary artery, along with initial ECGs showing subtle ST elevation in leads V2-V4, which then undergo dynamic changes alongside the Wellens pattern, typically indicating critical stenosis of the proximal LAD, represent a unique constellation of symptoms and findings.

In 1867, von Bezold and Hirt observed that injecting veratrum alkaloids led to a significant decrease in blood pressure, heart rate, and apnea [6]. Later, in 1915, Cramer noted similar effects with veratrum viride extracts in cats, as cited Dawes et al. [7]. In the late 1930s, Jarisch et al. studied cats injected with veratridine, confirming the reflex nature of these responses [8,9]. In 1947, Dawes et al. showed that reflex apnea was distinct from hemodynamic changes [10]. Over time, terms evolved, with the BJR now referring to bradycardia, vasodilation, and hypotension triggered by cardiac receptor stimulation [3]. The reflex originates in the cardiac sensory receptors mainly located in the inferoposterior wall of the left ventricle with the vagus nerve as the afferent pathway [11]. The efferent pathway involves sympathetic inhibition to peripheral vessels and parasympathetic stimulation to the heart via the vagus nerve [3]. The Bezold-Jarisch reflex can be triggered by conditions affecting the inferoposterior wall of the heart such as acute MI, myocardial ischemia, or intracoronary injection of contrast media [11]. Notably, the improvement in bradycardia following the administration of atropine, in our patient, suggests a vagally mediated response rather than direct ischemic injury to the sinoatrial node.

Approximately one-third of ACS cases in emergency departments may exhibit atypical symptoms, with specific demographic groups such as older individuals, females, diabetics, hypertensives, and those with heart failure being more prone [12]. Delayed diagnosis of ACS correlates with adverse outcomes and increased mortality, highlighting the necessity for increased awareness and consideration of atypical symptoms in ACS diagnosis and management. In our case, the patient's presentation aligns with the diagnostic challenges associated with ACS presentation. Despite chest pain being the hallmark symptom of ACS, our patient presented with bradycardia, hypotension, and syncope, attributed to the Bezold-Jarisch reflex. It is important to understand that the Bezold-Jarisch reflex is a known phenomenon that specifically occurs in the context of inferior wall myocardial ischemia or infarction due to ventricular mechanoreceptor stimulation [13].

The electrocardiogram (ECG) in our case displayed an important finding known as the type A Wellens pattern. The Wellens pattern is indicative of critical stenosis in the proximal LAD artery. The Wellens pattern typically manifests as biphasic T waves in the precordial leads (V2-V6) (type A, 25% of cases) or deeply and symmetrically inverted T waves (type B, 75% of cases) [14]. The Wellens pattern is often accompanied by minimal or no elevation in cardiac enzymes. This pattern signifies a high-risk condition, as patients presenting with it have a high likelihood of developing a large anterior wall MI in the near future [15,16]. Prompt recognition of the Wellens pattern on ECG is crucial, as it alerts clinicians to the presence of significant coronary artery disease and the urgent need for intervention to prevent a potential anterior wall MI and its associated complications.

The decision to prioritize revascularization of the proximal RCA over the LAD, despite the presence of the type A Wellens pattern on the ECG indicating critical stenosis in the LAD, was multifaceted. Angiographic evidence revealed a complete thrombotic lesion with 100% stenosis in the proximal RCA, posing an immediate threat due to its thrombotic nature and complete occlusion. Addressing this lesion first was crucial to prevent further myocardial damage and adverse outcomes. While the Wellens pattern suggested critical LAD stenosis, it did not mandate immediate intervention, especially considering that, angiographically, the distal LAD demonstrated 75% stenosis and IVUS assessment revealed a minimum luminal area of 3.5 mm². RCA revascularization was prioritized based on the clinical judgment that it would mitigate acute thrombotic risk and stabilize the patient's condition. This decision balanced the immediate thrombotic risk of the RCA lesion against the potential risk of an anterior wall MI associated with the Wellens pattern, ensuring patient safety and optimizing outcomes.

Interestingly, the absence of ST-segment elevation MI (STEMI) in this patient can be attributed to codominant coronary circulation, which likely provided collateral circulation and prevented complete occlusion of the affected artery. This highlights the importance of considering coronary anatomy variations in the pathophysiology of ACS presentations.

## Conclusions

This case illustrates a unique and complex instance of ACS, where the patient presented with the Bezold-Jarisch reflex, alongside critical coronary artery disease. It underscores the pivotal role of early recognition and intervention in navigating such clinical challenges and achieving favorable outcomes. By integrating clinical evaluation with advanced diagnostic modalities such as angiography and IVUS, we could tailor our management strategy effectively. Despite the presence of a type A Wellens pattern on the ECG, suggesting critical stenosis in the proximal LAD, the decision to prioritize revascularization of the proximal RCA was guided by angiographic evidence, revealing a complete thrombotic lesion with 100% stenosis, posing an immediate thrombotic risk. This illustrates the importance of a nuanced approach in weighing immediate thrombotic risks against potential adverse outcomes associated with atypical presentations of ACS. Ultimately, this case underscores the necessity for individualized management strategies and the integration of various diagnostic modalities to optimize patient care and outcomes in clinical practice.
